# Anti-Influenza Effect and Mechanisms of Lentinan in an ICR Mouse Model

**DOI:** 10.3389/fcimb.2022.892864

**Published:** 2022-05-20

**Authors:** Huan Cui, Cheng Zhang, Chunmao Zhang, Zhuming Cai, Ligong Chen, Zhaoliang Chen, Kui Zhao, Sina Qiao, Yingchun Wang, Lijia Meng, Shishan Dong, Juxiang Liu, Zhendong Guo

**Affiliations:** ^1^ Changchun Veterinary Research Institute, Chinese Academy of Agriculture Sciences, Changchun, China; ^2^ College of Animal Medicine, Jilin University, Changchun, China; ^3^ College of Veterinary Medicine, Hebei Agricultural University, Baoding, China

**Keywords:** lentinan, influenza virus, ICR mouse model, cytokine storm, TLR4/MyD88 signaling pathway

## Abstract

Influenza virus is a serious threat to global human health and public health security. There is an urgent need to develop new anti-influenza drugs. Lentinan (LNT) has attracted increasing attention in recent years. As potential protective agent, LNT has been shown to have anti-tumor, anti-inflammatory, and antiviral properties. However, there has been no further research into the anti-influenza action of lentinan *in vivo*, and the mechanism is still not fully understood. In this study, the anti-influenza effect and mechanism of Lentinan were studied in the Institute of Cancer Research (ICR) mouse model. The results showed that Lentinan had a high degree of protection in mice against infection with influenza A virus, delayed the emergence of clinical manifestations, improved the survival rate of mice, significantly prolonged the middle survival days, attenuated the weight loss, and reduced the lung coefficient of mice. It alleviated the pathological damage of mice infected with the influenza virus and improved blood indices. Lentinan treatment considerably inhibited inflammatory cytokine (TNF-α, IL-1β, IL-4, IL-5, IL-6) levels in the serum and lung and improved IFN-γ cytokine levels, which reduced cytokine storms caused by influenza virus infection. The underlying mechanisms of action involved Lentinan inhibiting the inflammatory response by regulating the TLR4/MyD88 signaling pathway. This study provides a foundation for the clinical application of Lentinan, and provides new insight into the development of novel immunomodulators.

## 1 Introduction

Influenza A is one of the major infectious diseases threatening human health, and the seasonal influenza epidemic has been widely studied (Kanekiyo et al., 2013; Hutchinson, 2018). It was reported that the influenza A virus (IAV) infects approximately 5% of adults and 20% of children worldwide every year, with up to 3 million severe cases and 250,000 deaths (Zhou et al., 2014; Fontana and Steven, 2015). According to the antigenicity of hemagglutinin (HA) and neuraminidase (NA) proteins, IAV can be categorized into many subtypes, such as H1N1, H3N2, H5N1, H5N6, H7N9, and H9N2 (Park and Ryu, 2018). Previous studies have shown that cross-species infection and transmission of avian influenza viruses such as H5N1, H5N6, H7N9, and H9N2 confer a broad spectrum of disease with varying severity and mortality, posing a serious threat to public health and human health (To et al., 2013; Zhang et al., 2016; Um et al., 2021). New strains of IAV with new pathogenicity and medication resistance evolve when HA and NA continue to mutate, resulting in seasonal influenza epidemics and localized outbreaks (Abdul-Careem et al., 2011; Perera and Peiris, 2015). Thus, there is an urgent need to develop more effective novel anti-influenza drugs.

IAV damages the body through viral replication and an excessive inflammatory response (Lobo et al., 2019). Infection with IAV can also stimulate the body to produce excessive cytokine, while a cytokine storm can cause acute lung injury (ALI) in the host, which is the main cause of mortality (Fukuyama and Kawaoka, 2011). Toll-like receptors (TLRs) are an important class of proteins involved in natural immunity and are also key receptors for the production of inflammatory mediators, among which TLR4 is closely related to pneumonia caused by IAV (Martin and Wurfel, 2008). MyD88 is a linker protein that plays an extremely important role in TLR signaling (Li et al., 2009). MyD88 recruits IL-1R-associated kinases (IRAKs) that activate TRAF-6 through autophosphorylation. TRAF-6 activates nuclear factor (NF)-κB, which induces related gene expression and secretes inflammatory cytokines (Downes and Marshall-Clarke, 2010). It has previously been shown that the activation of the TLR4 signaling pathway aggravates the lung damage caused by IAV (Imai et al., 2008; Nhu et al., 2010). Therefore, inhibition of the cytokine storm and regulation of the TLR4 signaling pathway are particularly important in the development of new anti-influenza drugs.

Lentinan (LNT) is a β-1,3-glucan extracted from Lentinus edodes that has good biological activity and its safety has also been affirmed (Mao et al., 2019; Kuang et al., 2021). LNT has immunomodulatory, anti-inflammatory, antiviral, and antitumor effects, and its good anti-inflammatory and antiviral effects have been widely recognized worldwide in recent years (Han et al., 2001; Ren et al., 2019; Chen et al., 2021; Yang et al., 2021; Du et al., 2022; Yang et al., 2022). According to studies, LNT can reduce inflammatory damage by reducing inflammatory cytokine release and can also control inflammation by inhibiting the TLR4 signaling pathway, thereby acting as an antiviral (Nishitani et al., 2013; Liu et al., 2019; Murphy et al., 2020). Based on these results, we speculate that LNT can treat influenza A. Oseltamivir is widely used as a neuraminidase inhibitor in the treatment of influenza A (Agrawal et al., 2010). Many studies on anti-influenza effects and mechanisms of drugs chose the ICR mouse model (Geng et al., 2019; Du et al., 2020; Yu et al., 2020; Cui et al., 2022). In our study, oseltamivir was used as a positive control drug to study the anti-influenza effect and mechanism of LNT in the ICR mouse model.

## 2 Materials and Methods

### 2.1 Ethics Statement

To ensure animal welfare, all research involving animals was approved by strict regulations. All the animal experiments were approved by the relevant regulatory agency of the Animal Ethical Committee of Changchun Veterinary Research Institute. (Document number of approval: SCXK202105099).

### 2.2 Animal Experiment and Sample Collection

#### 2.2.1 Animals and Grouping

A total of 120 male ICR mice weighing 18-20 g were purchased from Beijing Vital River Laboratory Animal Technology Co., Ltd. During the trial, ICR mice were fed using the IVC system and provided normal water and food. Sixty mice were randomly divided into six groups (10 mice per group). Six groups of mice were observed for clinical manifestations, survival rate, and 14-day continuous weight monitoring: the negative control group (control), the virus-infected group (model), the oseltamivir treatment group (oseltamivir), the lentinan low-dose treatment group (LNT-L), the lentinan medium-dose treatment group (LNT-M) and the lentinan high-dose treatment group (LNT-H). The other 60 ICR mice were grouped as above for sample collection and subsequent experimental testing.

#### 2.2.2 Source and Dosage of Drugs

Oseltamivir was purchased from Aladdin Bio-Chem Technology Co., Ltd. (Shanghai, China). Oseltamivir was diluted in phosphate buffer solution (PBS) at a concentration of 5 mg/mL and given intragastrically to ICR mice at a daily dose of 20 mg/kg, as previously reported (Tang et al., 2021). Lentinan was purchased from Solarbio Science & Technology Co., Ltd (Beijing, China. Lentinan dissolved in PBS was given to the LNT-L, LNT-M, and LNT-H groups by gavage at 5 mg/kg, 10 mg/kg, and 20 mg/kg, respectively (Kuang et al., 2021). At 2 hours postinfection, the mice were given drugs or PBS by oral gavage once a day for 5 days (Zhang et al., 2021).

#### 2.2.3 Virus and Infection

Influenza A virus A/PR/8/34 (H1N1) was stored by Changchun Veterinary Research Institute, Chinese Academy of Agriculture Sciences. The virus was amplified in 9-day-old specific-pathogen-free (SPF) chicken eggs (Beijing Boehringer Ingelheim Viton Biotechnology Co., Ltd). Then the harvested all-cystic fluid was inoculated into SPF chicken eggs and the 50% egg infectious dose (EID_50_) of the virus was determined. The 50% lethal dose (LD_50_) of A/PR/8/34 (H1N1) was determined in IRC mice. The EID_50_ and LD_50_ were calculated by the Reed - Muench method. Based on previous studies, the ICR mouse infection model was established (Reed and Muench, 1938; Wu et al., 2016; Ma et al., 2021a). In short, ICR mice were anesthetized with isoflurane and infected intranasally with a 50 μL virus suspension containing 2 LD_50_ viruses. The mice were randomly divided into 5 groups (n=10 for each group): the model group, oseltamivir group, LNT-L group, LNT-M group, and LNT-H group. Ten mice in the control group were also anesthetized with isoflurane and given 50 μL PBS as a control.

#### 2.2.4 Sample Collection

Experimental samples were collected from the mice of the six experimental groups 5 days postinfection (dpi). EDTA anticoagulant blood and ordinary nonanticoagulant blood were collected from mice under general anesthesia (isoflurane anesthesia). EDTA anticoagulant blood was used for routine blood testing. The normal non-anticoagulant blood was stored for 30 min at 37 °C, followed by centrifugation at 4,000 rpm for 10 min. Then, the serum was utilized to detect cytokines. The lungs of mice were collected and weighed to calculate the lung index. Each mouse’s lungs were divided into three parts. The first section was utilized to identify viral titers in mouse lungs. The second section was fixed in a 40% formaldehyde solution before being stained with hematoxylin and eosin (HE) and immunohistochemically. The RNA extracted from the last section of the lung was used for mRNA detection of cytokines and TLR4 signaling pathways.

### 2.3 Data Recording and Experimental Testing

#### 2.3.1 Clinical Manifestations, Survival Rate, and Body Weight

Six groups of mice were monitored continuously for 14 days after infection for clinical manifestations (reduced activity, back hair, tachypnea, etc.), survival, and body weight, to assess the protective effect by reducing survival time and mortality.

#### 2.3.2 Virus Titer and Lung Index

At 5 dpi, the lungs of each mouse were collected and weighed, and the lung index of the mice was calculated by the formula (Wei et al., 2018). The mouse lung samples were homogenized in 1 mL of PBS using a tissue lyser (Qiagen, Germany). Samples were clarified at 8,000 rpm at 4 °C for 10 min. The above-clarified lung homogenates were inoculated into SPF chicken eggs and the EID_50_ was determined by hemadsorption.

#### 2.3.3 Lung Histopathology

At 5 dpi, the lungs of each mouse were collected and fixed in 40% formaldehyde, and the samples were paraffin-embedded. The tissue samples were sectioned at a thickness of 4 μm and stained with hematoxylin and eosin. A light microscope was used to examine the pathological abnormalities in the lungs.

#### 2.3.4 Routine Blood Tests of Mice

Routine blood tests were performed using EDTA anticoagulant blood collected from mice at 5 dpi. The collected mouse blood was tested according to the manual of an automatic blood cell analysis instrument (BT-3200, Better, China). The differences in blood cells among different groups of mice were analyzed by the test results.

#### 2.3.5 Detection of the Cytokine and Signaling Pathways of TLR4

For cytokine detection, serum samples from mice taken at 5 dpi were used. The ELISA Kit was used according to the manufacturer’s instructions. Boster Biological Technology Co., Ltd (Wuhan, China). provided TNF-α (Item Number: EK0527), IL-1β (Item Number: EK0394), IL-4 (Item Number: EK0405), IL-5 (Item Number: EK0408), IL-6 (Item Number: EK0411), and IFN-γ (Item Number: EK0411) EK0375). To extract RNA from mouse lungs, an RNeasy Plus Universal Kit (73404, Qiagen, Germany) was employed. A Prime Script™ RT reagent Kit (RR037A, Takara, Dalian, China) was used for reverse transcription of the extracted RNA to obtain cDNA. Power SYBR™ Green PCR Master Mix was used for RT–qPCR of the obtained cDNA. The primers in **Table 1** were used to detect cytokines and TLR4 signaling pathway mRNA in mouse lungs. RT–qPCR was performed on an ABI 7500 Fast Real-Time PCR System (Thermo Fisher Scientific, USA). The RT-qPCR procedure was 50°C for 2 min and 95°C for 10 min. 40 cycles: 95°C for 15 sec and 60°C for 1 min. Data were analyzed using the 2^−ΔΔCt^ method (Pfaffl, 2001).

**Table 1:** Primer sequences for RT-qPCR.

Target Gene	Direction	Sequences (5’-3’)
TNF-α	Forward Reverse	CCCTCACACTCAGATCATCTTCT GCTACGACGTGGGCTACAG
IL-1β	Forward Reverse	GCAACTGTTCCTGAACTCAACT ATCTTTTGGGGTCCGTCAACT
IL-4	Forward Reverse	GGTCTCAACCCCCAGCTAGT GCCGATGATCTCTCTCAAGTGAT
IL-5	Forward Reverse	GCAATGAGACGATGAGGCTTC GCCCCTGAAAGATTTCTCCAATG
IL-6	Forward Reverse	TAGTCCTTCCTACCCCAATTTCC TTGGTCCTTAGCCACTCCTTC
IFN-γ	Forward Reverse	ATGAACGCTACACACTGCATC CCATCCTTTTGCCAGTTCCTC
TLR4	Forward Reverse	ATGGCATGGCTTACACCACC GAGGCCAATTTTGTCTCCACA
MyD88	Forward Reverse	AGGACAAACGCCGGAACTTTT GCCGATAGTCTGTCTGTTCTAGT
TRAF6	Forward Reverse	AAAGCGAGAGATTCTTTCCCTG ACTGGGGACAATTCACTAGAGC
NF-κB p65	Forward Reverse	AGGCTTCTGGGCCTTATGTG TGCTTCTCTCGCCAGGAATAC
GAPDH	Forward Reverse	AGGTCGGTGTGAACGGATTTG TGTAGACCATGTAGTTGAGGTCA

#### 2.3.6 Immunohistochemical Test

At 5 dpi, the lungs of each mouse were collected and fixed in 40% formaldehyde, and the samples were paraffin-embedded. The tissue samples were sectioned at a thickness of 4 μm, and the operation was carried out using the instructions of the two-step anti-rabbit IGG-HRP immunohistochemistry kit (SV0002, BOSTER, Wuhan, China). The primary antibodies used for immunohistochemistry were as follows: TLR4 (Cell Signaling, 14358S, 1:1000), MyD88 (Beyotime, AF2116, 1:100), TRAF6 (Cell Signaling, 67591S, 1:500), and phospho-NF-κB P65 (Cell Signaling, 3033S, 1:1000). The brown-positive products were observed under an optical microscope, and the integrated optical density (IOD) results were quantified as described using Image-Pro Plus 6.0 software (Media Cybernetics, USA) (Peng et al., 2016).

### 2.4 Statistical Analysis

The results are the means ± standard deviation (SD). Statistical differences were assessed by using the GraphPad Prism software version 8.0.2 (GraphPad Prism software, Inc San Diego, CA, USA) by one-way analysis of variance (ANOVA). P < 0.05 was deemed statistically significant.

## 3 Result

### 3.1 LNT Showed a Good Protective Effect on Infected Mice

**Figure 1:**
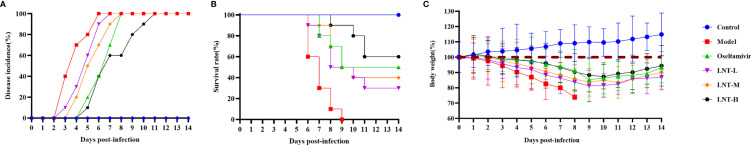
Protective effect of LNT on mice infected with influenza virus. Male ICR mice were intranasally infected with 2 LD_50_ influenza virus A/PR/8/34 (H1N1). At 2 hours post-infection, the mice were given drugs or PBS by oral gavage once a day for 5 days. Six groups (n=10) of mice were observed for clinical manifestations **(A)**, survival rate **(B)**, and weight monitoring **(C)** for 14 consecutive days.

The clinical manifestations, survival rates, and body weight changes of the six groups of mice (control, model, oseltamivir, LNT-L, LNT-M, and LNT-H) were monitored and recorded daily to determine the protective impact of LNT on mice infected with IAV (**Figure 1**). During the 14-day study period, the LNT therapy group exhibited good protection. At 3 dpi, mice in the model group displayed clinical signs such as reduced activity, erect back hair, and tachypnoea. However, clinical symptoms of the mice did not appear in the oseltamivir and LNT-H groups until 5 dpi, and LNT-L and LNT-M also delayed the emergence of clinical symptoms. Mice in the model group began to die at 6 dpi and all died at 9 dpi. At 7 dpi and 8 dpi, mice in the oseltamivir and LNT-H groups began to die, with death protection rates of 50% and 60%, respectively (**Table 2**). The death protection rates of LNT-L and LNT-M were 30% and 40%, respectively. We discovered that as the LNT dose increased, the protection rate of the mice also increased. Furthermore, middle survival days were prolonged in infected mice treated with LNT (**Table 2**). During the weight monitoring period, the model mice showed a continued drop in body weight until all died at 9 dpi, approximately 25%. The body weight of mice in the oseltamivir, LNT-L, LNT-M and LNT-H groups showed a trend of first decreasing and then increasing. Among the four groups, mice in the oseltamivir and LNT-H groups lost the least body weight and recovered most rapidly. The mice in the control groups were in good physical condition.

**Table 2:** The protective effect of Lentinan on infected mice.

Groups	Mice(n)	Death(n)	Death rate (%)	Death protective rate (%)	Middle survival days
Control	10	0	0	/	/
Model	10	10	100	/	7
Oseltamivir	10	5	50	50%	11.5
LNT-L	10	7	70	30%	9
LNT-M	10	6	60	40%	9.5
LNT-H	10	4	40	60%	>14

### 3.2 LNT Exerts a Protective Effect Against ALI

**Figure 2:**
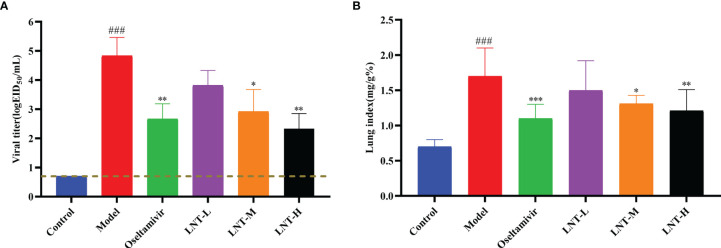
Protective effect of LNT on the lungs of mice infected with influenza virus. Male ICR mice were intranasally infected with 2 LD_50_ influenza virus A/PR/8/34 (H1N1). At 5 dpi, the lungs were harvested from the euthanized mice and weighed to calculate the lung index **(B)**. At the same time, the virus titer in the lungs was titrated with SPF chicken embryos **(A)**. ^###^p < 0.001, vs. control group (n=10); *p < 0.05, **p < 0.01, ***p < 0.001, vs. model group (n=10).

**Figure 3:**
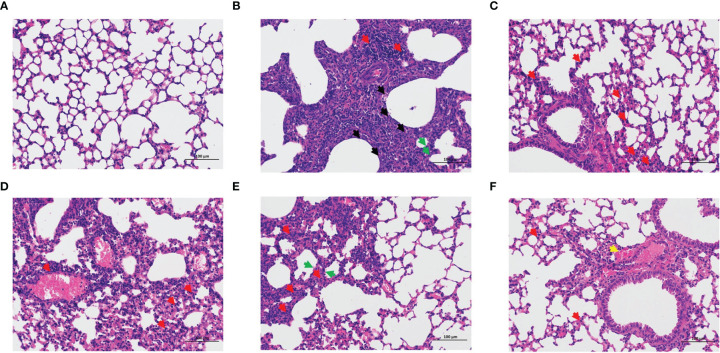
Pathological observation of the lung in mice. Lung pathological changes were observed by HE staining at 5 dpi (200 x): control group **(A)**, model group **(B)**, oseltamivir group **(C)**, LNT-L group **(D)**, LNT-M group **(E)**, and LNT-H group **(F)**. Necrosis of epithelial cells (black arrow); Inflammatory cell infiltration (red arrow); Alveolar wall thickening (green arrow); Bleeding (yellow arrow).

The lung coefficient and viral titer of mice at 5 dpi were determined to study the protective impact of LNT on ALI (**Figure 2**). The lung index (1.71 ± 0.39) and virus titer (10^4.83 ± 0.63^ EID_50_/mL) in the model group were significantly higher than those in the control group (P < 0.001). The pulmonary coefficient and virus titer of the four treatment groups (oseltamivir, LNT-L, LNT-M, and LNT-H) were all reduced to some extent compared to the model group, indicating that the pulmonary edema of mice had been improved to some extent, with the oseltamivir and LNT-H groups having the most obvious improvement effect. HE staining of the lungs of mice was performed to further study the protective impact of LNT on ALI in mice (**Figure 3**). The control group showed no obvious pathological damage, whereas the model group showed alveolar thickening, inflammatory cell infiltration, and epithelial cell death. In the four therapy groups, lung damage was reduced, with oseltamivir and LNT-H providing the best protection.

### 3.3 LNT Improved the Blood Indeces of Mice Infected With IAV

**Figure 4:**
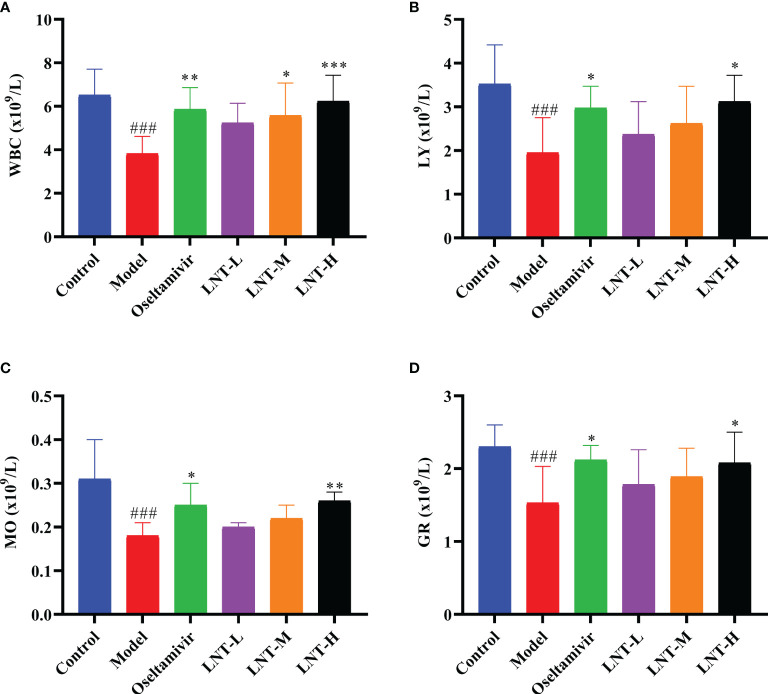
Routine blood results of mice. At 5 dpi, EDTA anticoagulant blood was collected for routine blood testing. An automatic blood cell analyzer (BT-3200, Better, China) was used to detect the mouse samples. The blood cell data are shown in the figure. **(A)** White blood cells; **(B)** lymphocytes; **(C)** monocytes; **(D)** granulocytes. WBC stands for white blood cells, LY for lymphocytes, MO for monocytes and GR for granulocytes. ^###^p < 0.001, vs control group (n=10); *p < 0.05, **p < 0.01, ***p < 0.001, vs. model group (n=10).

The blood routine of mice was measured to evaluate the influence of LNT on blood indices in mice infected with IAV. Routine blood data are an important indicator of inflammation caused by the IAV. In this study, four important indicators of routine blood tesys (white blood cells, lymphocytes, monocytes, and granulocytes) were analyzed (**Figure 4**). These four types of blood cells were found in much lower numbers in the model group than in the control group. Compared to control mice, the four treatment groups (oseltamivir, LNT-L, LNT-M, and LNT-H) were effective in inhibiting the decrease in the four blood cell types in mice, with oseltamivir and LNT-H showing the best inhibitory effect.

### 3.4 Effects of LNT on Cytokines in Infected Mice

**Figure 5:**
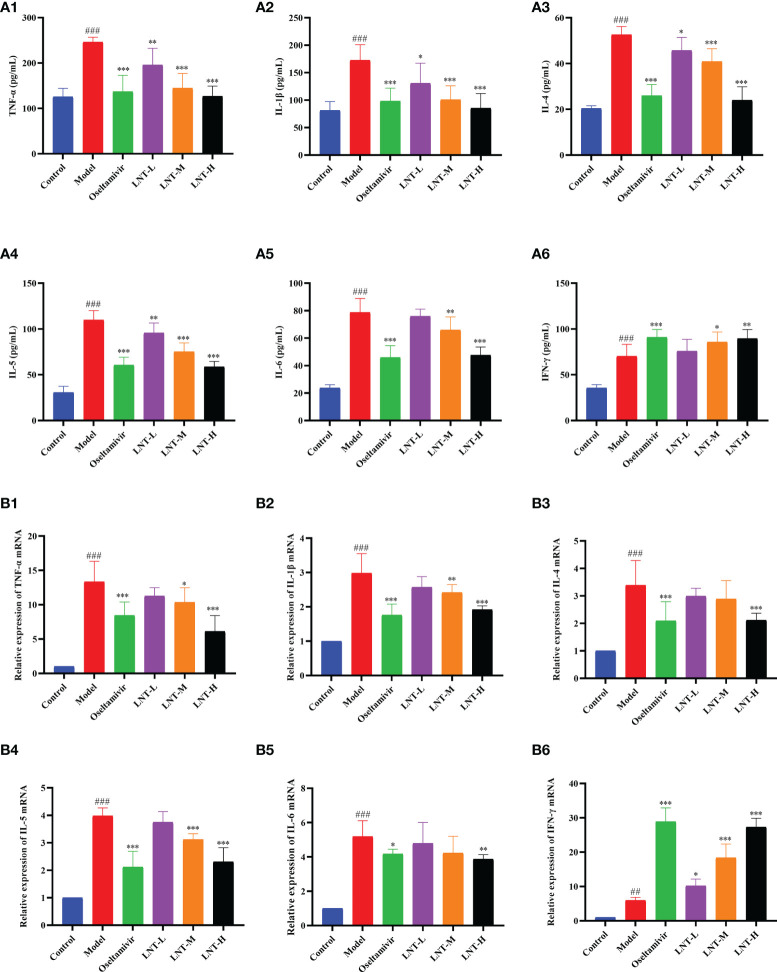
Effects of LNT on cytokines in mice. The serum and lungs of 5 dpi mice were collected. Cytokines in the serum of mice were detected by ELISA method, and cytokines in the lungs of mice were detected by RT–qPCR. LNT decreased the levels of TNF-α, IL-1β, IL-4, IL-5 and IL-6 in the serum **(A)** and lung **(B)** of mice, and increased the level of IFN-γ. ^###^p < 0.001, ^##^p < 0.01, vs. the control group (n=10); *p < 0.05, **p < 0.01, ***p < 0.001, vs. the model group (n=10).

During IAV infection, inflammation-related cytokines increase significantly, and cytokine storms are thought to be a major cause of death (Fukuyama and Kawaoka, 2011; Labella and Merel, 2013). The quantities of cytokines in mouse serum and the mRNA expression of cytokines in mouse lungs were examined in this study. TNF-α, IL-1β, IL-4, IL-5, IL-6, and IFN-γ expression levels in the serum of mice in the model group were significantly greater than those in the control group (P<0.001), as shown in **Figure 5A**. TNF-α, IL-1β, IL-4, IL-5, and IL-6 expression in serum was inhibited to varying degrees in the four treatment groups (oseltamivir group, LNT-L group, LNT-M group, and LNT-H group) compared to the model group. Serum IFN-γ levels, on the other hand, were significantly elevated, with the oseltamivir and LNT-H having the greatest influence on serum cytokines. The mRNA expression trend of these six cytokines in the lungs of mice was the same as that in the serum (**Figure 5B**). The regulation of LNT on cytokine expression in the serum and lung of mice was improved when the LNT dosage was increased.

### 3.5 Inhibition of LNT on TLR4/MyD88 Signaling Pathway by LNT

**Figure 6:**
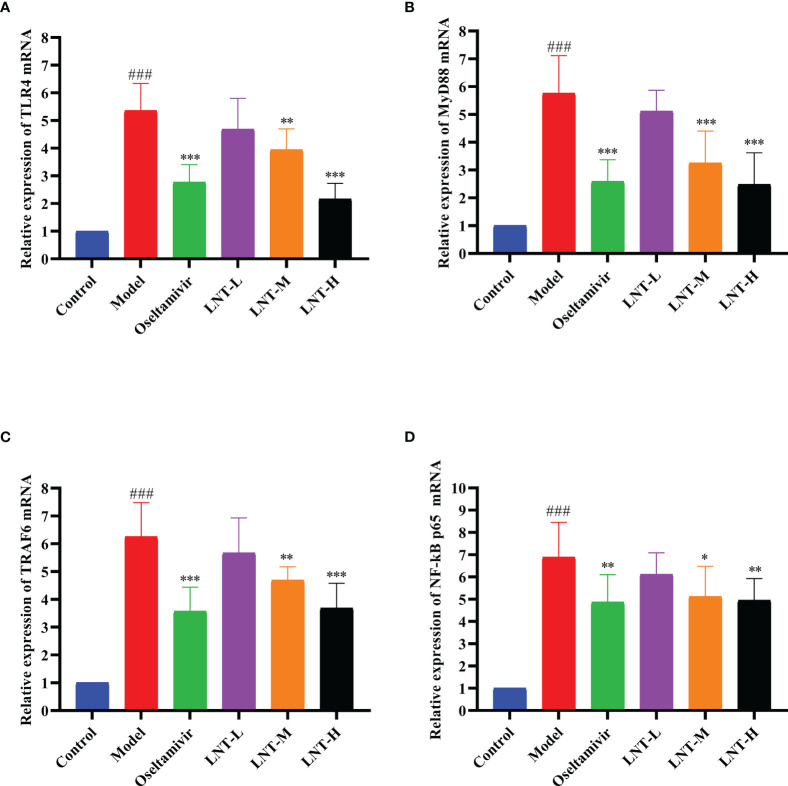
Effect of LNT on the TLR4 signaling pathway in the mouse lung. The lungs of 5 dpi mice were collected, and the mRNA levels of TLR4 **(A)**, MyD88 **(B)**, TRAF6 **(C)** and NF-κB P65 **(D)** in the TLR4 signaling pathway were detected by RT–qPCR. LNT inhibited the expression levels of the four mRNAs. ^###^p < 0.001, vs control group (n=10); *p < 0.05, **p < 0.01, ***p < 0.001, vs. model group (n=10).

**Figure 7:**
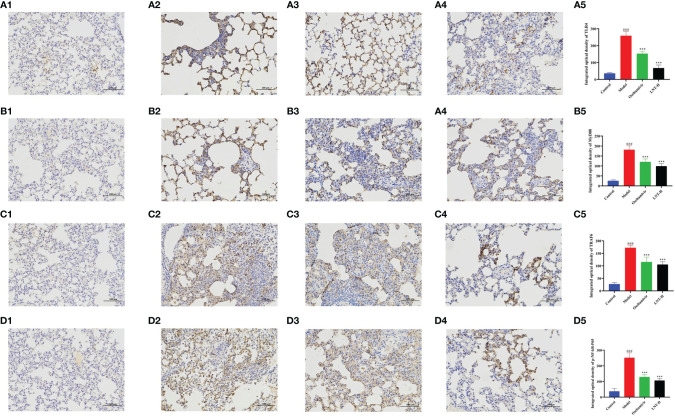
Immunohistochemical staining observation of key proteins in the TLR4 signaling pathway in mouse lung tissue. Lung tissues of 5 dpi mice were collected and the expression levels of TLR4 **(A)**, MyD88 **(B)**, TRAF6 **(C)** and P-NF-κB P65 **(D)** in the TLR4 signaling pathway were quantitatively analyzed by immunohistochemical staining (200 x). A1, B1, C1, and D1 represent the control group; A2, B2, C2, and D2 represent the model group, A3, B3, C3, and D3 represent oseltamivir group, A4, B4, C4, and D4 represent the LNT-H group; and A5, B5, C5, and D5 represent the IOD quantification results of brown positive products. ^###^p < 0.001, vs control group (n=10); ***p < 0.001, vs. model group (n=10).

TLR4, MyD88, TRAF6, and NF-B P65 mRNA expression levels were evaluated by RT–qPCR in this work to assess the effects of LNT on the TLR4/MyD88 signaling pathway (**Figure 6**). The expression levels of TLR4, MyD88, TRAF6, and NF-κB P65 mRNA in the model group were significantly upregulated compared with those in the control group (P < 0.001). Compared with the model group, the four treatment groups (oseltamivir group, LNT-L group, LNT-M group, and LNT-H group) exhibited varied inhibitory effects on the mRNA expression levels of TLR4, MyD88, TRAF6, and NF-κB P65 in the lungs of mice. Except for the LNT-L group, there were significant differences in the other three groups (P < 0.05). TLR4, MyD88, TRAF6, and NF-B P65 mRNA expression levels in the lungs of mice were significantly reduced in the oseltamivir and LNT-H groups. Immunohistochemical techniques were utilized to evaluate the protein expression levels of TLR4, MyD88, TRAF6, and P-NF-B P65 in the TLR4/MyD88 signaling pathway. The two treatment groups with the best therapeutic effect (oseltamivir group and LNT-H group) were chosen for immunohistochemistry staining after a thorough evaluation of all other findings in this study. To present the data, we tallied the IOD of all immunohistochemical staining sections and derived the mean values and SD (**Figure 7**). The protein expression levels of TLR4, MyD88, TRAF6, and P-NF-κB P65 in the model group were significantly upregulated compared with those in the control group (P < 0.001). The protein expression levels of TLR4, MyD88, TRAF6, and P-NF-κB P65 in the lungs of both treatment groups (oseltamivir and LNT-H) were significantly downregulated compared with those in the model group (P < 0.001).

## 4 Discussion

IAV is a pathogen that can cause a pandemic and seriously threatens human health and public health security all over the world. As IAV continues to evolve and mutate, new drug-resistant strains are emerging, making the development of new anti-influenza medications critical. LNT has received much attention in recent years, and the safety of LNT in food has been proven (Sun et al., 2020). LNT has a variety of actions, including immunomodulatory, anti-inflammatory, antitumor, and antiviral activities, according to recent research (Han et al., 2001; Ren et al., 2019; Gu et al., 2022; Liu et al., 2022). Previous studies have reported that LNT possesses anti-influenza activity, while the anti-influenza mechanism of LNT is still unclear (Irinoda et al., 1992). In recent research, LNT has been proven to have a favorable immunomodulatory impact *in vitro* and is suspected of having a regulatory influence on cytokine storms induced by a new coronavirus and IAV, but this has yet to be confirmed *in vivo* (Murphy et al., 2020). There has been no further research into the anti-influenza action of LNT *in vivo*, and the mechanism is still not fully understood.

In this study, LNT showed a good protective effect on mice infected with IAV. It delayed the appearance of clinical manifestations and the appearance of the first dead mouse (**Figure 1**), improved the death protection rate of mice, significantly prolonged middle survival days (**Table 2**), and reduced weight loss. In comparison to the oseltamivir group, the LNT-H group’s protective impact on mice was more noticeable. LNT not only protected infected mice from infection but also helped prevent animals from ALI. LNT lowered not only the virus titer in mouse lungs, but also the lung coefficient, reducing the pathological harm produced by IAV infection in mice. Our study on the anti-influenza activity of LNT *in vivo* is more extensive than a previous study (Irinoda et al., 1992). Routine blood tests are critical in the early detection of IAV infection, with the four white blood cell markers of lymphocytes, monocytes, and granulocytes being particularly essential (Shaim et al., 2020). In mice infected with IAV, drug therapy has been demonstrated to enhance blood indicators (Chik et al., 2004). Our findings are consistent with prior findings, and infected mouse blood indices can be dramatically improved after treatment with LNT.

Host defense systems have been demonstrated to alter cytokine levels and the onset of clinical symptoms of IAV in previous investigations (Hayden et al., 1998; Kaiser et al., 2001). Although cytokines can aid in pathogen clearance, unregulated cytokine release can result in cytokine storms (Loo and Gale, 2007). The etiology of severe influenza is linked to cytokine storms as well as direct damage from viral multiplication in the lungs (Fukuyama and Kawaoka, 2011; To et al., 2013; Lobo et al., 2019). Therefore, immunomodulators combined with anti-inflammatory therapy may be a viable strategy to treat drug-resistant IAV infections in the future (Peiris et al., 2009). LNT has been identified as a good immunomodulator in numerous anti-inflammatory and antiviral studies (Mao et al., 2019; Li et al., 2020; Kuang et al., 2021). TNF-α, IL-1β, IL-4, IL-5, IL-6, and IFN-γ are the key cytokines that have been implicated in the inflammatory response following IAV infection. TNF-α is a multifunctional inflammatory cytokine that plays a key role in the development of severe influenza. TNF-α levels are linked to the severity of infection-induced pulmonary damage (Aldridge et al., 2009). Overexpression of IL-4, IL-5, and IL-6, on the other hand, was linked to illness severity (Damjanovic et al., 2011; Wei et al., 2018; Ma et al., 2021b). L-1β is a major inflammatory factor that contributes to ALI and plays a significant role in the development of ALI (Niu et al., 2019). IFN-γ has been found to reduce early death of IAV infection in mice, and it plays a key role in virus clearance (Wiley et al., 2001; Weiss et al., 2010; Kawahara et al., 2015). In this study, the LNT significantly reduced TNF-α, IL-1β, IL-4, IL-5, and IL-6 levels, improved IFN-γ levels, and alleviated cytokine storm caused by IAV infection. Recent research has discovered that elevated plasma levels of IL-1β and IL-6 expression are a typical sign of the cytokine storm generated by new coronavirus pneumonia (COVID-19) (Liu et al., 2020; Zhang et al., 2020). LNT, on the other hand, has strong regulatory effects on IL-1β and IL-6, and some researchers have postulated that it may also regulate COVID-19 (Murphy et al., 2020). Thus, scientists should examine whether LNT has a regulatory influence on COVID-19.

**Figure 8:**
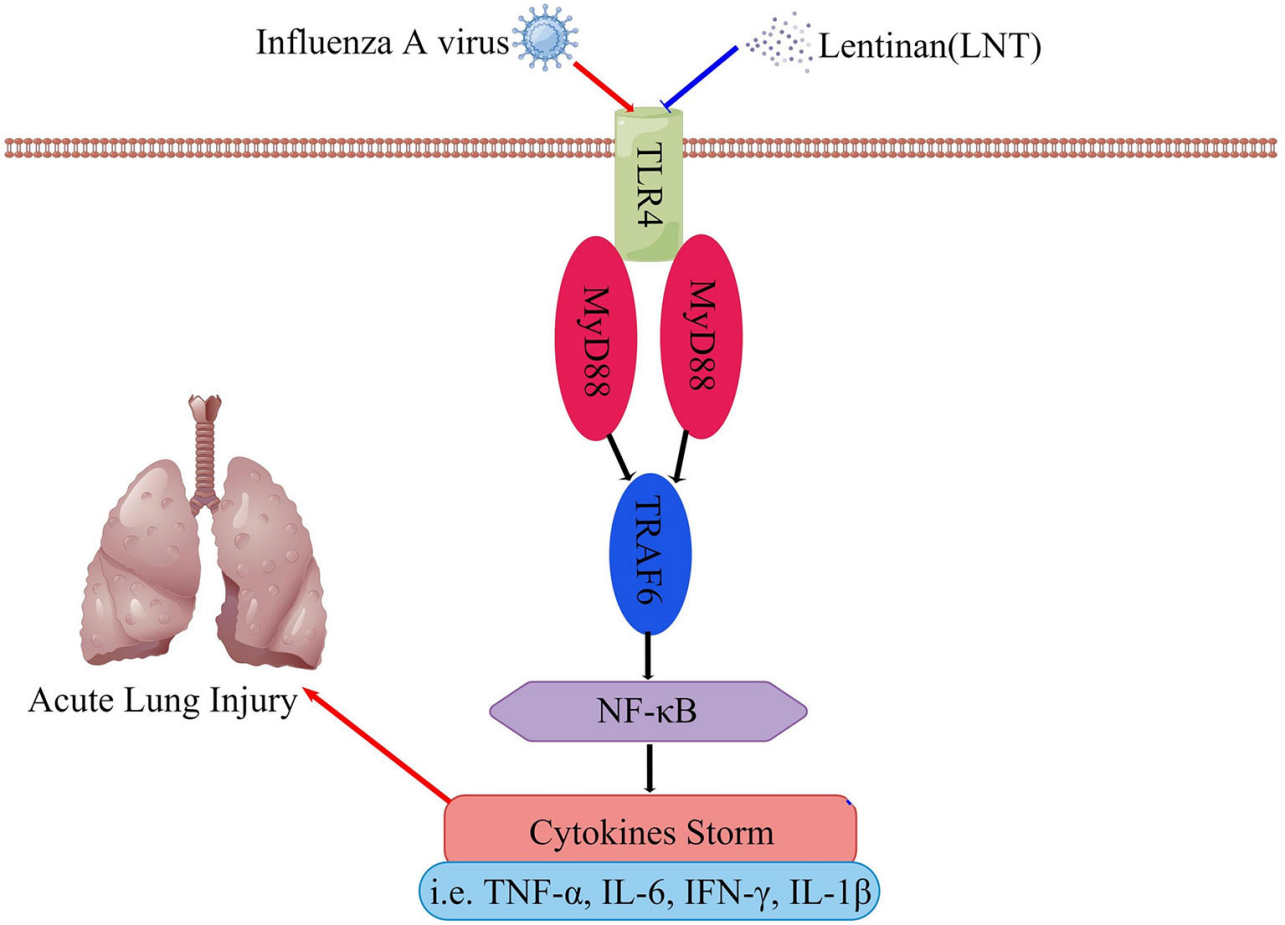
LNT regulates the cytokine storm through the TLR4 signaling pathway and alleviates acute lung injury caused by influenza virus infection. This figure was drawn on Figdraw.

TLR4 is involved in the inflammatory response to IAV infection, and activation of the TLR4 signaling pathway exacerbates the lung damage induced by the virus (Imai et al., 2008; Martin and Wurfel, 2008; Nhu et al., 2010). In the TLR4 signaling pathway, which is necessary for cytokine generation, MyD88 serves as a vital bridge protein (Yamamoto et al., 2003; Deguine and Barton, 2014). TRAF-6 triggers NF-κB, which causes gene expression and inflammatory cytokine secretion (Downes and Marshall-Clarke, 2010). There are many members of TLRs, including TLR3, TLR7, etc. In our study, no other TLRs were found to play an important role in the anti-influenza process of lentinan. LNT regulates sepsis *via* the TLR4/MyD88 signaling pathway, according to previous research (Li et al., 2020; Kuang et al., 2021). The TLR4/MyD88 signaling pathway was investigated using RT–qPCR and immunohistochemistry in this study. Our findings revealed that LNT reduces the severity of pneumonia induced by IAV infection by modulating the TLR4/MyD88 signaling pathway, resulting in a more effective therapeutic impact (**Figure 8**).

In conclusion, our findings reveal that LNT can reduce ALI in IAV-infected mice, and we studied the mechanism of LNT action by inhibiting the inflammatory response by controlling the TLR4/MyD88 signaling pathway. This research laid the groundwork for the clinical use of LNT and the development of novel immunomodulators.

## Data Availability Statement

The original contributions presented in the study are included in the article/supplementary material. Further inquiries can be directed to the corresponding authors.

## Ethics Statement

The animal study was reviewed and approved by The Animal Ethical Committee of Changchun Veterinary Research Institute.

## Author Contributions

JL, SD, and ZG designed the project. CZ, HC, and CMZ performed the experiments. CMZ, LC, ZC, KZ, SQ, YW, snd LM analyzed the data. CZ, ZG, and HC drafted the manuscript. CMZ, JL, and SD critically revised the manuscript. All authors contributed to the article and approved the submitted version.

## Funding

This study was supported by the National Natural Science Foundation of China (82150202), Key Research Projects in Hebei Province (18227517D) and Hebei Industrial Technology System (HBCT2018150210).

## Conflict of Interest

The authors declare that the research was conducted in the absence of any commercial or financial relationships that could be construed as a potential conflict of interest.

## Publisher’s Note

All claims expressed in this article are solely those of the authors and do not necessarily represent those of their affiliated organizations, or those of the publisher, the editors and the reviewers. Any product that may be evaluated in this article, or claim that may be made by its manufacturer, is not guaranteed or endorsed by the publisher.
